# Supplementary material for: From sequence to enzyme mechanism using multi-label machine learning
Source: BMC Bioinformatics. 2014 May 19;15:150. doi: 10.1186/1471-2105-15-150 (PMC4229970; doi:10.1186/1471-2105-15-150)
Supplement: Additional file 2 — Java code of ml2db. Additional file ml2db_code.tar.gz contains the Java source code to run the multi-label machine learning experiments and save the results to database. The code’s Javadoc is included. [file 1471-2105-15-150-S2.zip › additional file 2/ml2db/ml2db/doc/main/ml2db/al/utility/pickers/byfrequency/package-use.html]

Uses of Package main.ml2db.al.utility.pickers.byfrequency


JavaScript is disabled on your browser.


- Overview
- Package
- Class
- Use
- Tree
- Deprecated
- Index
- Help

- Prev
- Next

- Frames
- No Frames

- All Classes

# Uses of Package main.ml2db.al.utility.pickers.byfrequency

- Packages that use main.ml2db.al.utility.pickers.byfrequency

  | Package | Description |
  | --- | --- |
  |  |  |
  | --- | --- |
  | main.ml2db.al.utility.pickers.byfrequency |  |
  | test.ml2db.al.utility.neighbourhood |  |
- Classes in main.ml2db.al.utility.pickers.byfrequency used by main.ml2db.al.utility.pickers.byfrequency

  Class and Description

  ByFrequencyPicker

  Picks each of the features (class, attribute or attribute set) by descending
  order of frequency, and then, uniformly randomly, one of the instances of
  that feature.
- Classes in main.ml2db.al.utility.pickers.byfrequency used by test.ml2db.al.utility.neighbourhood

  Class and Description

  NearestNeighboursByFreqUtility

  Class

- Overview
- Package
- Class
- Use
- Tree
- Deprecated
- Index
- Help

- Prev
- Next

- Frames
- No Frames

- All Classes
